# Emergency Response to Tizanidine Overdose: A Case Report on Critical Care Strategies

**DOI:** 10.7759/cureus.70696

**Published:** 2024-10-02

**Authors:** Marwa Morgom, Shahzad Anjum, Moayad A Elgassim, Sujood Awadelseed, Leena Saeed

**Affiliations:** 1 Emergency Medicine, Hamad Medical Corporation, Doha, QAT; 2 Medical Education, Hamad Medical Corporation, Doha, QAT; 3 Medical Research Center, Hamad Medical Corporation, Doha, QAT

**Keywords:** bradycardia, critical care, elderly population, tizanidine overdose, tizanidine poisoning

## Abstract

A 75-year-old woman with multiple comorbidities presented with altered mental status, bradycardia, and hypotension following the ingestion of tizanidine. Emergency medical services (EMS) promptly initiated pacing and administered vasopressors, leading to the patient’s recovery to her baseline condition after receiving supportive care. ECG findings indicated pre-existing cardiac abnormalities. She was admitted to the intensive care unit for further observation and management and was later discharged in stable condition. This case underscores the potential for severe adverse effects of tizanidine and highlights the critical need for timely recognition and intervention.

## Introduction

Sinus bradycardia, characterized by a heart rate below 60 beats per minute while maintaining a sinus rhythm, can significantly impair cardiac output and oxygen delivery to vital organs. Although commonly linked to electrolyte imbalances, cardiac conditions, or endocrine disorders, bradycardia can also be induced by pharmacologic agents. Tizanidine, an alpha-2 adrenergic agonist used primarily to manage muscle spasticity, has been reported to cause bradycardia, though such occurrences are relatively infrequent [1]. 

Tizanidine's therapeutic action involves the inhibition of presynaptic neurotransmitter release, which leads to reduced sympathetic tone and subsequent decreases in both blood pressure and heart rate. The drug is subject to extensive first-pass metabolism and possesses a relatively short half-life, although variations in individual drug metabolism can affect its pharmacokinetics [2]. 

Tizanidine is frequently linked to adverse effects such as sedation, dizziness, and dry mouth. However, bradycardia is also another potential side effect that may occur. The potential for tizanidine-induced bradycardia, particularly in individuals with predispositions or when used in conjunction with other bradycardia-inducing medications, highlights the necessity for vigilant patient monitoring and thorough risk assessment [3]. 

This case report examines a severe instance of tizanidine-induced bradycardia and hypotension, emphasizing the critical management approaches required to address this potentially life-threatening adverse effect. 

## Case presentation

A 75-year-old female patient with multiple comorbidities, including a history of coronary artery disease and end-stage renal disease (ESRD) on dialysis, was brought to our center by ambulance; she had a reduced level of consciousness and was found to have bradycardia and hypotension. The patient’s family reported that she self-administered an overdose of tizanidine (24 mg) as an over-the-counter medication for leg pain caused by painful diabetic neuropathy in both limbs, after which the symptoms started. 

EMS found her with bradycardia and hypotension. Heart rate (HR) was 30, and blood pressure (BP) was unrecordable by EMS (Table 1). EMS started pacing at rate 70 with electrical capture at 105mA. The rate increased to 90 beats per minute. She received IV norepinephrine and IV fluids and subsequently recovered with a BP of 94/39; GCS improved to 15/15 after pacing. Adrenaline infusion started at 5 mcg/min during transport. The adrenaline was decreased to 2 mcg/min and stopped prior to handover.

**Table 1:** Vital signs recorded by EMS and on arrival to the ED. EMS: emergency medical services; ED: emergency department

Vital	Initially recorded by EMS	At ED
Pulse rate (beats per minute)	30	90 with pacing
Respiratory rate (breaths per minutes)	20	13
SpO2	92%	97%
Systolic BP (mmHg)	unrecordable	160
Diastolic BP (mmHg)	unrecordable	58

**Figure 1:**
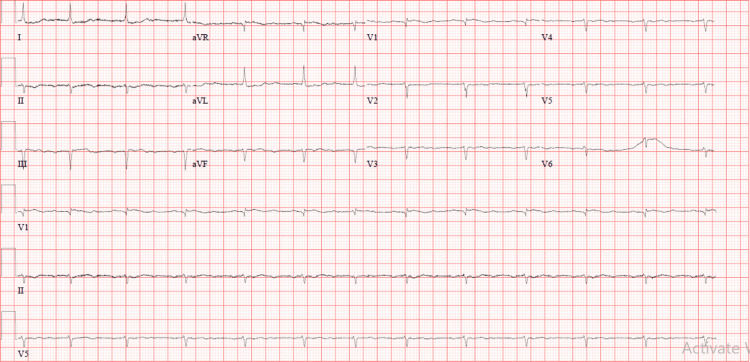
ECG upon arrival to ED. ECG: electrocardiogram; ED: emergency department

Upon arrival at the emergency department (ED), the patient experienced dizziness, depression, and loss of consciousness, her HR was 90 beats per minute with pacing along with intermittent hypotension in between (Table 1). The ECG showed normal sinus rhythm (NSR) with a prolonged PR interval, poor R wave progression from V1 to V6, and T-wave inversion in the lateral leads (indicative of old changes) (Figure 1).

She was given a dopamine infusion (200 mg) along with supportive measures for blood pressure and HR until the tizanidine effect was weaned off. Laboratory investigations revealed high prothrombin time (PT), high activated partial thromboplastin time (APTT), high creatinine, and high alkaline phosphatase (Table 2).

**Table 2:** Results of laboratory investigation done in the emergency department. WBC: white blood cells; RBC: red blood cells; Hgb: hemoglobin; Hct: hematocrit; MCV: mean corpuscular volume; MCH: mean corpuscular hemoglobin; MCHC: mean corpuscular hemoglobin concentration; RDW-CV: red cell distribution width-coefficient of variation; MPV: mean platelet volume; INR: international normalized ratio; APTT: activated partial thromboplastin time; ALT: alanine aminotransferase; AST: aspartate aminotransferase; NT pro-BNP: N-terminal pro-B-type natriuretic peptide; troponin-T HS: troponin-T high sensitivity

Parameters	Labs at ED	Normal ranges
WBC	9	4x10^3 -10x10^3/uL
RBC	4 x10	3.8x10^6 -4.6x10^6 u/L
Hgb	12.7	12-15 gm/dL
Hct	41.4	36–46%
MCV	103	83 - 101 fL
MCH	31.6	27-32 pg
MCHC	30.7	31.5–34.5 gm/dL
RDW-CV	14.3	11.6-14 %
Platelet	177 x10	150x10^3 - 410x10^3 u/L
MPV	10.5	9.6-12 fL
Prothrombin	14.4	9.4-12.5 seconds
INR	1.3	<1.1
APTT	37	25.1–36.5 seconds
Urea	6.9	2.5-7.8 mmol/L
Creatinine	279	44-80 umol/L
Sodium	133	133-146 mmol/L
Potassium	4.2	3.5-5.3 mmol/L
Chloride	101	95-108 mmol/L
Bicarbonate	20	22-29 mmol/L
Calcium	2.03	2.2 to 2.7 mmol/L
Adjusted calcium	2.45	2.2-2.6 mmol/L
Bilirubin T	8	0-21 umol/L
Total protein	60	60-80 gm/L
Albumin	19	35-50 gm/L
Alkaline phosphatase	265	35-104 U/L
ALT	14	0-33 U/L
AST	32	0-33 U/L
NT pro-BNP	22717	<125 pg-mL
Troponin-T HS	71	3-10 ng/L
Glucose	9.1	3.3-5.5 mmol/L
CRP	12.7	0-5 mg/L

**Figure 2:**
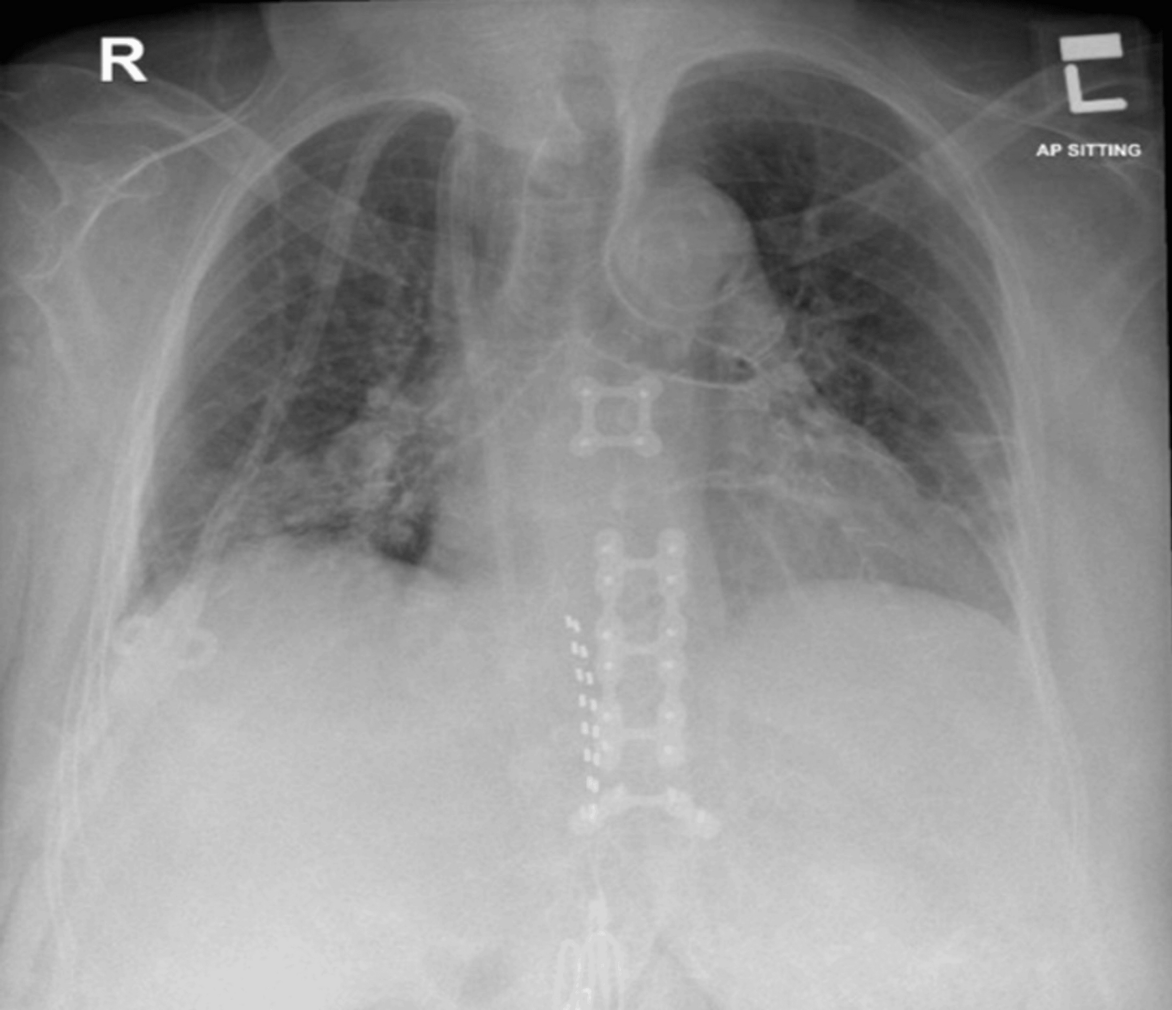
Antero-posterior sitting chest x-ray showing both lung fields with prominent broncho-vascular markings with bibasal infiltrates.

The initial chest X-ray shows prominent broncho-vascular markings with bibasilar infiltrates in both lung fields (Figure 2). When she was transferred to MICU, she was noted to be oriented, not in distress, and vitally stable in response to dopamine 1.5mg with a Glasgow Coma Scale (GCS) score of 15/15 (Table 3).

**Table 3:** Vital signs in the MICU. MICU: medical intensive care unit; BP: blood pressure

Vitals at MICU	Readings	Normal references in adults
Pulse rate	84	60-100 beats per minute
Respiratory rate	16	12-18 breaths per minute
SpO2	98	95-100%
Systolic BP	131	90-120 mmHg
Diastolic BP	61	60-80 mmHg

**Figure 3:**
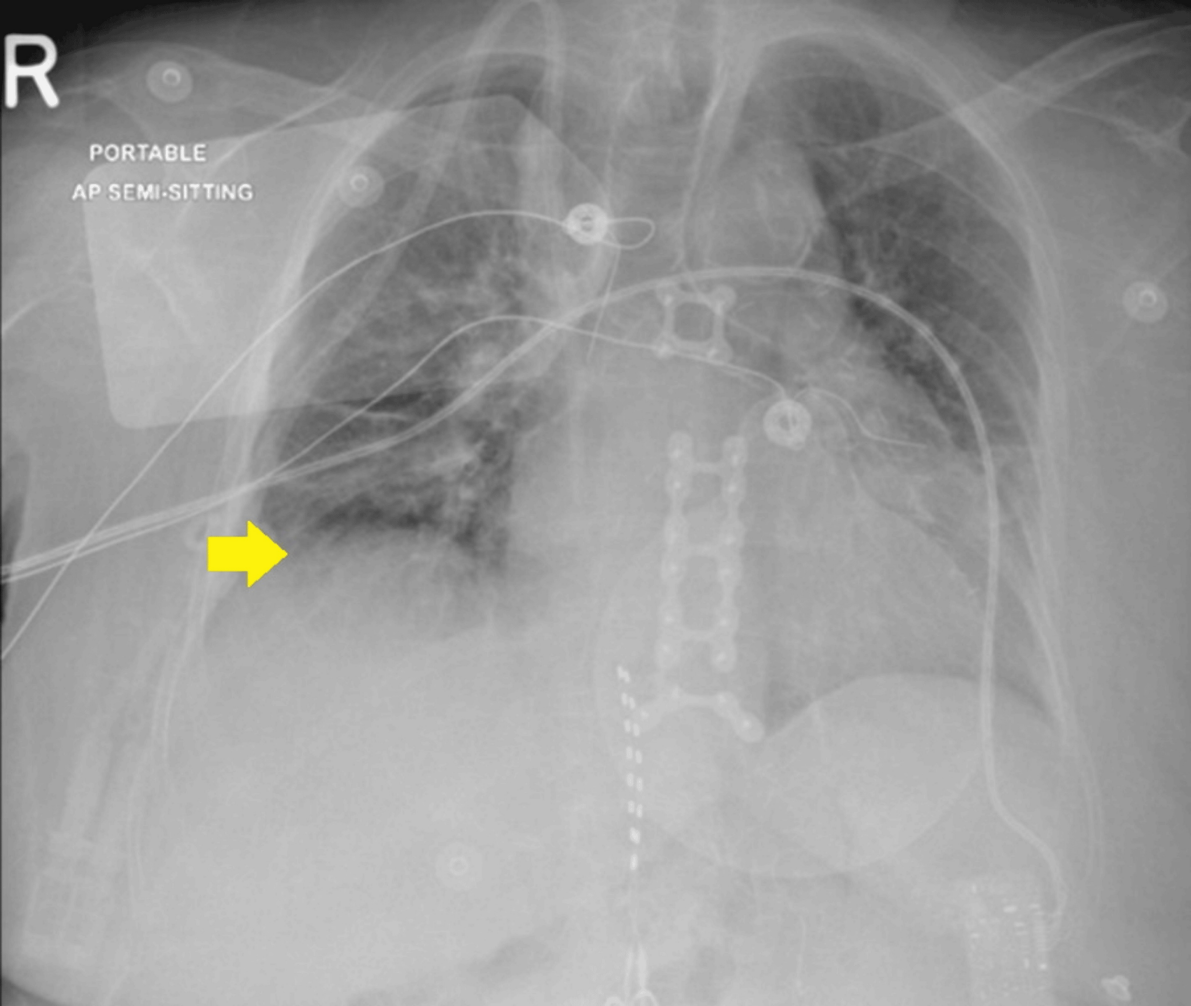
Semi-sitting chest X-ray findings Compared to the previous radiograph, there is interval placement of defibrillator pads over the chest wall. Streaky opacity can be noted in the right lower zone (yellow arrow).

The management plan continued, and dopamine was tapered down and stopped. The repeated chest X-ray in comparison to the previous radiograph, shows interval placement of defibrillator pads over the chest wall, with streaky opacity noted in the right lower zone, allowing for the differences in positioning and technique. No other significant interval changes were observed (Figure 3). She was subsequently discharged from MICU two days after her arrival at the emergency department. 

## Discussion

Sinus bradycardia, characterized by a sinus rhythm with a heart rate below 60 beats per minute, can be clinically significant if the reduced heart rate impairs the delivery of oxygenated blood to tissues. It's essential to evaluate bradycardia thoroughly to rule out various underlying causes such as electrolyte imbalances, cardiac diseases, hypothyroidism, elevated intracranial pressure, sick sinus syndrome, and hypothermia [4,5]. Tizanidine, a drug used to manage muscle spasticity, has been associated with bradycardia, often seen with PR interval prolongation on an ECG. 

Tizanidine is an anti-spastic medication that provides similar efficacy to baclofen while generally having a better tolerability profile. It was first approved by the FDA in 1996 and functions as an alpha-2 adrenergic receptor agonist. The drug reduces spasticity by inhibiting the release of excitatory neurotransmitters pre-synaptically, which decreases neuronal firing and muscle spasms. Additionally, it lowers norepinephrine release, which leads to a reduction in blood pressure and heart rate [6]. Tizanidine is subject to substantial first-pass metabolism, leading to an oral bioavailability of roughly 40% and a half-life of approximately 2.5 hours. It is primarily broken down by the liver enzyme CYP1A2 and is predominantly eliminated through the kidneys within two to four hours following ingestion [6]. However, genetic variations can influence the activity of CYP1A2, leading to variability in drug metabolism among individuals [7,8]. 

Commonly reported side effects of tizanidine include dry mouth (46-50%), somnolence (46-50%), dizziness (16-20%), asthenia (10-45%), blurred vision (3%), constipation (4%), infection (6%), elevated liver transaminases (3-5%), speech disorders (3%), vomiting (3%), urinary frequency (3%), urinary tract infections (10%), as well as bradycardia, hallucinations, and gastrointestinal disturbances [9,10]. Instances of bradycardia induced by tizanidine at standard doses are infrequently reported in PubMed searches. 

Tizanidine-induced bradycardia has been documented in cases involving concurrent use of CYP1A2 inhibitors, occasionally leading to the requirement for a permanent pacemaker [11]. However, reports of bradycardia occurring with a low dose of tizanidine (2 mg) in the absence of drug interactions, metabolic disorders, structural cardiac conditions, or abrupt cessation of smoking an action known to decrease CYP1A2 enzyme activity potentially are exceptionally uncommon [12]. 

One case involved a patient who experienced sinus bradycardia with a heart rate of 30 beats per minute after taking 2 mg of tizanidine daily for two months to manage nocturnal shoulder muscle pain. The ECG showed significant bradycardia, necessitating a pacemaker [13]. Given this context, there have been no prior reports of severe symptomatic bradycardia resulting from a single dose of tizanidine in the absence of concomitant medications that could elevate the risk of symptomatic bradycardia.

A review of the patient’s medication history revealed no use of cytochrome P450-modifying drugs that could alter the clearance of tizanidine, nor were any bradycardia-inducing medications, such as calcium channel blockers or antiarrhythmics, in use. Although a beta-blocker had been prescribed previously, it had never been utilized by the patient. Considering these considerations, tizanidine should be used with caution in elderly patients, and comprehensive screening for potential drug interactions is advised [11]. 

## Conclusions

This case report describes a patient who experienced a pronounced and potentially life-threatening drop in heart rate and blood pressure following the administration of tizanidine. While tizanidine is known to reduce blood pressure and heart rate by diminishing sympathetic nervous system activity, this case highlights an unusually severe reaction to the drug. 

The discussion points to the significance of evaluating the patient's medical history, including underlying conditions that may predispose individuals to such adverse effects. The report also emphasizes the importance of vigilant monitoring for patients at higher risk for tizanidine-related side effects, especially the elderly population, and the need for timely intervention in cases of severe bradycardia and hypotension. 
